# Lyophilized Nasal Inserts of Atomoxetine HCl Solid Lipid Nanoparticles for Brain Targeting as a Treatment of Attention-Deficit/Hyperactivity Disorder (ADHD): A Pharmacokinetics Study on Rats

**DOI:** 10.3390/ph16020326

**Published:** 2023-02-20

**Authors:** Mahmoud H. Teaima, Merhan Taha El-Nadi, Raghda Rabe Hamed, Mohamed A. El-Nabarawi, Rehab Abdelmonem

**Affiliations:** 1 Department of Pharmaceutics and Industrial Pharmacy, Faculty of Pharmacy, Cairo University, Cairo P.O. Box 11562, Egypt; 2 Department of Pharmaceutics, Egyptian Drug Authority (EDA), Giza P.O. Box 12511, Egypt; 3 Industrial Pharmacy Department, College of Pharmaceutical Sciences and Drug Manufacturing, Misr University for Science and Technology, Cairo P.O. Box 12566, Egypt

**Keywords:** ADHD, atomoxetine HCl, brain targeting, solid lipid nanoparticles (SLNs), nasal inserts

## Abstract

The study aims to investigate the ability of lyophilized nasal inserts of nanosized atomoxetine HCl solid lipid nanoparticles (ATM-SLNs) to transport atomoxetine (ATM) directly to the brain and overcome the first-pass metabolism. In this case, 16 formulae of (ATM-SLNs) were prepared using hot melt emulsification, stirring and ultrasonication method technique. A full factorial design was established with 2^4^ trials by optimization of four variables; lipid type (Compritol 888 ATO or stearic acid) (X1), lipid to drug ratio [(1:2) or (2:1)] (X2), span 60: Pluronic f127 ratio [(1:3) or (3:1)] (X3) and probe sonication time (five or ten minutes) (X4). The prepared SLNs were characterized for entrapment efficiency (EE%), in-vitro drug release after 30 min (Q30min), particle size (PS), zeta potential (ZP) and polydispersity index (PDI). Design Expert^®^ software was used to select the optimum two formulae. The morphological examination for the optimum two formulae was carried out using a transmission electron microscope (TEM). Furthermore, eight lyophilized nasal inserts were prepared by using a 2^3^ full factorial design by optimization of three variables: type of (ATM-SLNs) formula (X1), type of polymer (NOVEON AA1 or HPMC K100m) (X2) and concentration of polymer (X3). They were evaluated for nasal inserts’ physicochemical properties. The two optimum inserts were selected by Design Expert^®^ software. The two optimum insets with the highest desirability values were (S4 and S8). They were subjected to DSC thermal stability study and in-vivo study on rats. They were compared with atomoxetine oral solution, atomoxetine (3 mg/kg, intraperitoneal injection) and the pure atomoxetine solution loaded in lyophilized insert. (ATM-SLNs) showed EE% range of (41.14 mg ± 1.8% to 90.6 mg ± 2.8%), (Q30min%) of (27.11 ± 5.9% to 91.08 ± 0.15%), ZP of (−8.52 ± 0.75 to −28.4 ± 0.212% mV), PS of (320.9 ± 110.81% nm to 936.7 ± 229.6% nm) and PDI of (0.222 ± 0.132% to 0.658 ± 0.03%). Additionally, the two optimum (ATM-SLNs) formulae chosen, i.e., F7 and F9 showed spherical morphology. Nasal inserts had assay of drug content of (82.5 ± 2.5% to 103.94 ± 3.94%), Q15min% of (89.9 ± 6.4% to 100%) and Muco-adhesion strength of (3510.5 ± 140.21 to 9319.5 ± 39.425). DSC results of S4 and S8 showed compatibility of (ATM) with the other excipients. S8 and S4 also showed higher trans-nasal permeation to the brain with brain targeting efficiency of (211.3% and 177.42%, respectively) and drug transport percentages of (52.7% and 43.64%, respectively). To conclude, lyophilized nasal inserts of (ATM-SLNs) enhanced (ATM) trans-nasal drug targeting permeation and brain targeting efficiency.

## 1. Introduction

Attention-Deficit/Hyperactivity Disorder (ADHD) is a highly heritable ailment and considered one of the most common neurodevelopmental disorders of childhood that was reported to affect 84.7 million people worldwide according to 2019 survey [1,2]. Children with ADHD usually have difficulty in paying attention, controlling impulsive behavior and acting without thoroughly considering expected results. The major parts of the brain showing dysfunction in (ADHD) are the prefrontal cortex, caudate nucleus, and cerebellum [3]. The network activity between these regions is achieved by the regulation of the two neurotransmitters, dopamine (DA), and norepinephrine (NE) acting in conjunction with each other [4].

Atomoxetine (ATM), a non-stimulant, selective norepinephrine reuptake inhibitor, is indicated orally for treating ADHD in children above 6 years of age and adults. Although atomoxetine has high water solubility and intestinal permeability (BCS Class I) drug, it has bioavailability of 63% in extensive metabolizers and 94% in poor metabolizers [5].

Targeted drug delivery of atomoxetine to the brain can be achieved via the olfactory nerve which constitutes the first cranial pair. Axons of the olfactory sensory neurons integrate the olfactory nerve, allowing the nasal cavity and brain to communicate without any relay [6]. In addition to allowing direct access to the brain, nasal drug delivery circumvents the blood brain barrier, bypasses the hepatic first-pass metabolism, achieves non-invasive drug delivery and hence increases the bioavailability of the drug [7,8]. Furthermore, in order to cross the blood brain barrier (BBB), a drug molecule must be lipid-soluble and have a molecular weight of less than 400 Da [9].

Solid lipid nanoparticles (SLNs) consist of a colloidal carrier system which is made of a solid matrix of high-melting lipids incorporated in the core and coated by the drug and physiologically compatible hydrophilic surfactants. Small particle size (50 to 1000 nm), biocompatibility, self-assembling capabilities, ability to cross the blood-brain barrier, and cost-effectiveness make SLNs unique in the world of nanotechnology. Solid lipids commonly used are of fatty acid origin (e.g., stearic acid), triglycerides (e.g., tristearin,) and steroids (e.g., cholesterol). Compritol 888 ATO was selected as the lipid carrier owing to its distinguished features of a high amount of mono-, di-, and triglycerides that help the drug to be incorporated into the lipid matrix of solid lipid nanoparticles, as well as being of accepted Generally Recognized As Safe (GRAS) status excipient. Moreover, Compritol 888 ATO was used because of its low hydrophilic-lipophilic balance (HLB) value of 2 which would increase the lipophilicity of the hydrophilic drug and subsequently increases its permeation through the blood-brain barrier (BBB) to the brain. Stearic acid (HLB = 14.9) was also used to compare between the two lipids in respect of the effect of HLB value of each lipid, the molecular weight and the branching of the structure of the solid lipid on the particle size, in-vitro drug release, entrapment efficiency of the resulting solid lipid nanoparticles [10,11], as well as the effect of the two lipids on the drug deposition in the brain [12,13]. Solid lipids were also stabilized by using a physiologically-compatible surfactant such as polyvinyl alcohol (PVA) and a mixture of co-surfactants, e.g., Span 60 and Pluronic F127. The use of surfactants decreases the agglomeration of nanoparticles produced in the lipid dispersion system [14].

Lyophilization of SLNs can increase the stability of the lipid dispersion by decreasing their hydrolysis over long-term storage [15,16]. Lyophilized nasal inserts of atomoxetine-loaded solid lipid nanoparticles were fabricated from two types of polymers, e.g., hydroxypropyl methylcellulose (HPMC-K100m) or NOVEON AA1 (Polycarbophil USP) that have high viscosity and excellent muco-adhesion properties to the nasal mucosa. Therefore, overcoming the mucociliary clearance of the drug from the nose. They also act as matrix forming agents in the formulation of the solid inserts [17]. Glycine can also be used as a collapse-protecting agent in the formulation of the nasal inserts [18].

The study was conducted to demonstrate the ability of atomoxetine to cross the blood-brain barrier upon incorporation of atomoxetine into solid lipid nanoparticles. Solid lipid nanoparticles were stabilized by freeze-drying technique to prevent their degradation. Lyophilized nasal inserts were fabricated from two types of polymers, i.e., hydroxypropyl methylcellulose and NOVEON. They were also characterized for their mucoadhesion strength, in vitro drug release and residual water content after lyophilization. After nasal administration, Atomoxetine HCl in plasma and brain of rats were determined by LC-MS/MS system and compared with the marketed oral solution, intravenous drug solution and lyophilized nasal insert dosage form of pure drug solution [19].

## 2. Results and Discussion

### 2.1. Evaluation of the Prepared ATM-Loaded SLNs

#### 2.1.1. Entrapment Efficiency (EE%)

**Figure 1:**
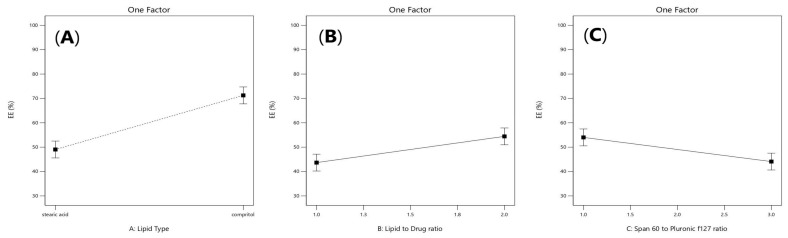
Linear plot of main effect of lipid type (X1) (**A**), lipid to drug ratio (X2) (**B**), and span 60 to pluronic F127 ratio (X3) (**C**) on entrapment efficiency (EE%).

**Figure 2:**
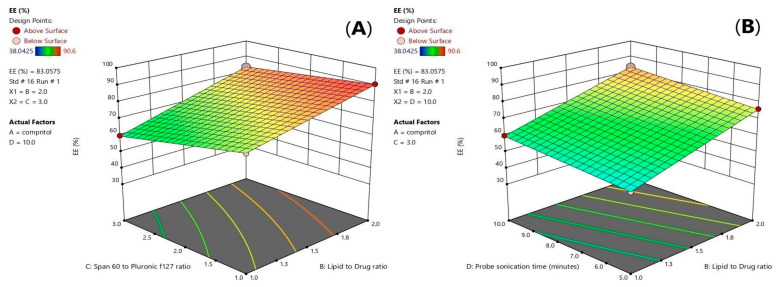
3D-diagram of effect of three factor interaction (3FI) of both (BC) interaction (**A**) and (BD) interaction (**B**) on entrapment efficiency (EE%). Note: B, lipid to drug ratio; C, span 60 to pluronic F127 ratio, and D, probe sonication time.

Values of the entrapment efficiency (EE%) were ranged 41.14 ± 1.8% to 90.5725 ± 2.8%, as demonstrated in Table 1. Lipid Type (X1) had a significant effect on EE% (*p* = 0.0001). Compritol 888 ATO showed the highest EE%, because of the complex nature of Compritol that was attributed to the long chain fatty acid (C22) that is attached to mono-, di-, and triacylglycerols lead to less perfect orientation, leaving more space for the drug to be loaded and enhancing the solubilization and incorporation of the drug into the lipid core [20]. As shown in Figure 1C, it was also found that ATM-loaded SLNs contained higher ratio of Pluronic F127 to Span 60 had a significant effect (*p* = 0.001) and showed the highest EE% due to the high HLB value of Pluronic F127 (HLB = 22) that was able to entrap more of the hydrophilic drug in the core of the solid lipid nanoparticles [21,22]. Increasing lipid concentration significantly increased the amount of the entrapped drug (*p* = 0.007). It was detected that EE% decreased by decreasing the amount of lipid from 36 to 9 mg which could be associated with the reduction in the surface area available for the drug to be loaded into solid lipid nanoparticles as well as, increasing lipid concentration would increase the stability of the nanoparticles and decreasing expulsion of the drug to the external media. As shown in Table 2, the statistical ANOVA analysis additionally revealed that the probe sonication time (X4) had a significant *p*-value of 0.0044. Furthermore, clear positive synergistic interactions had been noticed between the two factors (X1) and (X2) (*p*-value = 0.0042) and the two factors (X1) and (X4) (*p*-value = 0.0339). Additionally, ANOVA analysis of the EE data confirmed the significance of the model (*p*-value = 0.0003). As shown in Figure 2A,B, clear positive synergistic interaction had been noticed between the two factors (B) and (C) and the two factors (B) and (D), respectively. Moreover, the predicted R^2^ of (0.8793) was in reasonable agreement with the adjusted R^2^ of (0.9646), the difference is less than 0.2. adequate precision value equals 21.4 confirming the model validity to navigate the design space.

#### 2.1.2. Particle Size (PS)

**Figure 3:**
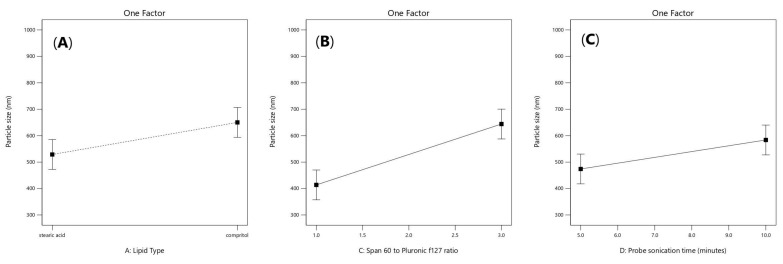
Linear plot of main effect of lipid type (X1) (**A**), span 60 to pluronic F127 ratio (X3) (**B**) and probe sonication time (X4) (**C**) on particle size (PS).

Particle size values of (ATM- loaded SLNs) formulations ranged from 320.9 ± 110.81 to 936.7 ± 229.6 nm as demonstrated in Table 1. The factors that had a significant effect on the particle size according to the ANOVA test results, are the lipid type (X1), span 60 to Pluronic f127 ratio (X3) and the probe sonication time (X4). As shown in Figure 3A, it was observed that the formulae prepared from stearic acid had significantly smaller particle size values than the formulae prepared from Compritol 888 ATO (*p* = 0.035), which could be related to the chemical composition of glyceryl dibehenate (i.e., Compritol ATO 888) which is a mixture of diacylglycerols, together with monoacylglycerols and triacylglycerols in addition to the long alkyl chain of dibehenic acid (C22). On the other hand, stearic acid is composed of a saturated chain of 18 carbon atoms [23]. Moreover, the higher amount of drug entrapped in Compritol-based SLNs lead to formation of larger particle size SLNs. It was reported that there is a direct relationship between particle size and EE% as the particle size depends on the distance between the SLNs bilayers, which is increased because of the inclusion of more drug molecules within the lipid core [24]. As shown in Figure 3B, a direct relationship was observed between the particle size enlargement and span 60 (HLB = 4.7) to Pluronic f127 (HLB = 22) ratio; (*p* = 0.0010), as Pluronic F127 have significant ability to incorporate hydrophilic drugs and self- assemble into small nanosized solid lipid nanoparticles [25]. As shown in Figure 3C, it was also found that increasing the probe sonication time (from 5 min to 10 min) had negative effect on particle size reduction (*p* = 0.022). The transformation of the solid lipid (Compritol 888 ATO) due to lipid polymorphism during longer probe sonication time had led to aggregation and increase in size of nanoparticles along with the expulsion of drug molecules incorporated in lipid nanoparticles to the external media [26,27]. Moreover, upon increasing the amount of Compritol 888 ATO in the SLN formulae simultaneously caused an increase in particle size that was related to the tendency of lipid to coalesce at high concentrations along with the fact that increasing lipid amount provides more space for drug molecules to be entrapped [28].

#### 2.1.3. Zeta Potential and Polydispersity Index

**Figure 4:**
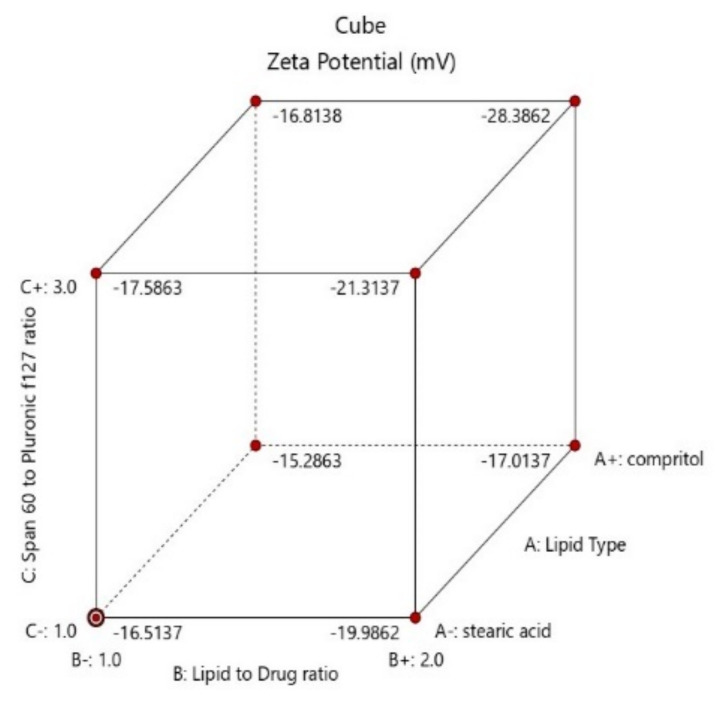
Cube diagram of effect of lipid type (X1), lipid to drug ratio (X2) and span 60 to Pluronic f127 ratio (X3) and on zeta potential (ZP).

As shown in Table 1, zeta potential values of the prepared formulae ranged from—8.53 to–28.4 mV, whereas PDI values ranged from 0.222 ± 0.132 to 0.611 ± 0.04. Atomoxetine-loaded SLNs formulae containing stearic acid had significantly higher negative ZP and lower PDI than the formulae containing Compritol 888 ATO according to the ANOVA test results (*p* = 0.0143 and 0.0552 for effect of lipid type (X1) on ZP and PDI, respectively). The higher negative ZP is attributed to the negative charge of stearic acid obtained from the carboxylic acid moiety oriented in the hydrophilic surface of nanoparticles [29]. As shown in Figure 4, it was also found that using lower co-surfactant ratio of span 60: Pluronic f127 of (1:3) enhanced the stability of the formed pre-emulsion and decreased the tendency of the nanoparticles to aggregate (*p*-value = 0.0053) [24]. Moreover, the negative surface charges were also obtained from the negative groups present in the polar head of span 60, Pluronic f127, PVA and stearic acid [30]. Furthermore, increasing the lipid to drug ratio (X2) was found to increase the stability of the nanoparticles (*p*-value = 0.0052). Increasing probe sonication time to 10 min (X4) led to an increase in the zeta potential (*p*-value = 0.0088).

#### 2.1.4. Study of the In-Vitro Release Profile of ATM-Loaded SLNs

**Figure 5:**
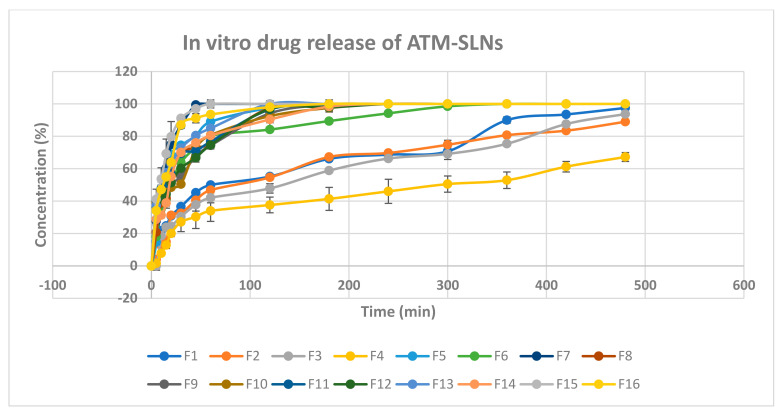
In vitro drug release chart of atomoxetine HCl solid lipid nanoparticles.

Figure 5 shows the in-vitro released percentages of Atomoxetine from its prepared 16 solid lipid nanoparticles formulae. It ranged between 27.11 ± 5.9% to 91.08 ± 0.15% after 30 min (Q30min) and ranged also between 67.25 ± 2.7% to 100 ± 0% after 8 h (Q8h%). Taking the lipid type into consideration, Compritol 888 ATO gave higher percentage of drug release than Stearic acid. Compritol 888 ATO is composed of a mixture of mono-, di-, and triacylglycerols of di-behenic acid which produced higher drug load per each SLNs and thereafter higher driving force for drug release [31]. 

ANOVA analysis of the in-vitro release data after 30 min detected the significance of the model (*p*-value = 0.0004). Additionally, the predicted R2 (0.8713) obtained was in a good agreement with the adjusted R2 (0.9623) with difference less than 0.2. The adequate precision of the model was 21.141. The coded equation representing the relationship between the independent variables and the in vitro drug release after 30 min values was as follows:

In vitro release in 30 min = 60.28 + 14.13 X1 − 7.38X2 + 5.86X3 + 2.65X4 + 5.26X1X2 + 3.36 X1X3 − 0.9X1X4 − 3.43X2X3 + 1.95X2X4 + 2.46X3X4.

**Figure 6:**
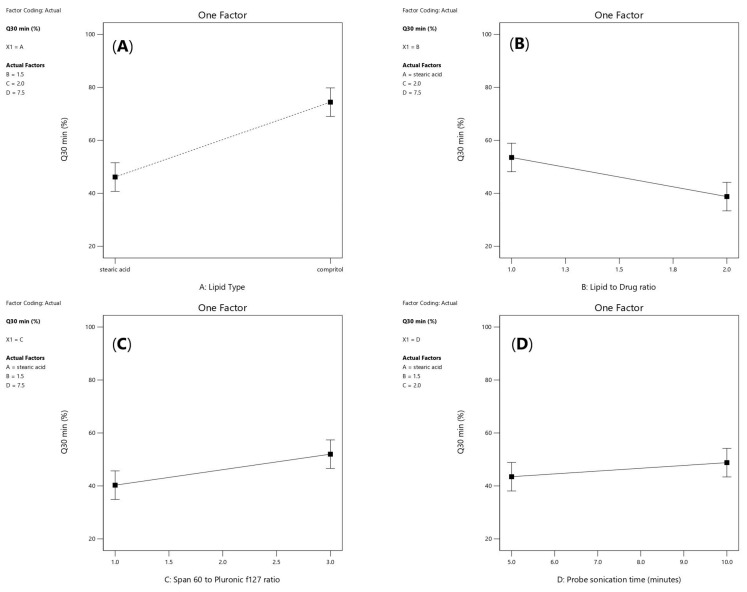
Linear plot of main effect of lipid type (X1) (**A**), lipid to drug ratio (X2) (**B**), span 60 to pluronic F127 ratio (X3) (**C**) and probe sonication time (X4) (**D**) on Q30min (%). Note: Q30min (%); percentage of drug released after 30 min.

As shown in Figure 6A, it was noticed that lipid type (X1) had a significant effect on the amount of drug released after 30 min (*p* < 0.0001). Additionally, lipid to drug ratio (X2) had also a significant effect (*p* = 0.0006) as illustrated in Figure 6B. Moreover, span 60: PF127 ratio was significant (*p* =0.0017) and probe sonication time (X4) was significant (*p* = 0.0387) as presented in Figure 6C and Figure 6D, respectively.

**Figure 7:**
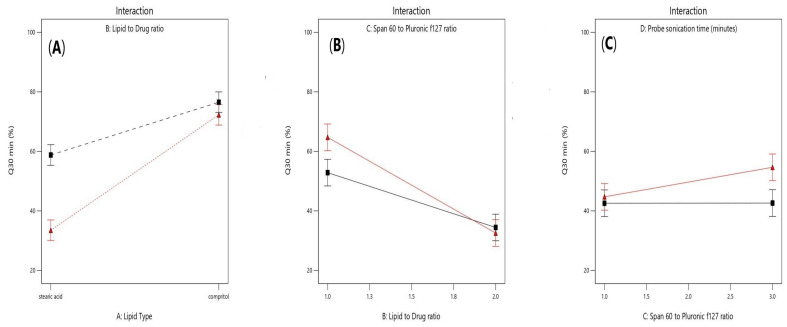
Effect of interaction between lipid type (X1) and lipid to drug ratio (X2) (**A**), lipid to drug ratio (X2) and span 60 to Pluronic F127 ratio (X3) (**B**), and span 60 to pluronic F127 (X3) and probe sonication time (X4) (**C**) on Q30min (%).

As shown in Figure 7A, a positive interaction had been noticed between the two factors (X1) and (X2) (*p*-value = 0.0027). Furthermore, as shown in Figure 7B, a significant negative interaction was observed between increasing lipid to drug ratio (X2) and increasing span 60 to Pluronic F127 ratio (*p* = 0.0155) because the HLB value of Span 60 is 4.7 while Pluronic F127 have HLB value of 22, so upon decreasing the amount of Pluronic F127 and increasing lipid concentration, the drug release will be hindered. Moreover, a positive significant interaction was observed between increasing probe sonication time (X4) and increasing span 60 to Pluronic f127 ratio (X3) (*p* = 0.0492), as shown in Figure 7C as increasing probe sonication time (10 min) will decrease the particle size of SLNs as well as increasing span 60 (3.75 mg/mL) to Pluronic F127 (1.25 mg/mL) ratio produced smaller vesicles owing to the larger structure of Pluronic F127 which constitutes of polyoxyethylene-polyoxypropylene triblock copolymer of general formula E 106 P 70 E 106, with an average molar mass of 13,000 [32,33].

#### 2.1.5. Kinetic Analysis of In-Vitro Release Data

Mathematical models were used to predict the release mechanism of the prepared solid lipid nanoparticles formulations and to compare their release profile. Various release kinetic models were tested, including zero order (direct relationship between cumulative percentages of drug released and time), first order (direct relationship between the log cumulative percentages of drug remaining and time), diffusion (direct relationship between cumulative percentages released and the square root of time) kinetic models, and Korsmeyer Peppas kinetic model (if n < 0.5 fickian diffusion).The regression coefficient (r2) was used as an indicator of the best fit for each of the considered models. As shown in Table 3, F1, F7–F9, F11–F16 formulations followed fickian diffusion model of drug release while, F2–F6 and F10 followed diffusion model of drug release.

#### 2.1.6. Optimization of the Solid Lipid Nanoparticles Formulations

The optimization criteria were set to minimize particle size, and Q30min and maximize entrapment efficiency (EE%) and zeta potential for selection of the optimum formula. As shown in Table 1, the formulae giving the highest numerical desirability were F7 and F9 with a value of 0.654 and 0.628, respectively. As illustrated in Table 3, the predicted responses for those formulations were particle size values of 801.52 and 374.89 nm, ZP of −28.4 and −16.51 mV, EE% of 83.29 and 54.97%, Q30min of 86.75 and 54.96%, respectively, whereas the actual responses were PS of 795.60 and 392.10 nm, ZP of −28.39 and −16.5 mV, EE% of 83.06 and 55.20%, Q30min of 84.25 and 51.98%, respectively. The results showed the high similarity between the observed and the predicted values of the two optimum formulae. Hence, F7 and F9 ATM-loaded SLNs can be considered as a promising SLN formulations. Therefore, they were selected for further investigations.

#### 2.1.7. Transmission Electron Microscopy (TEM) of the Optimized SLNs Formulations

**Figure 8:**
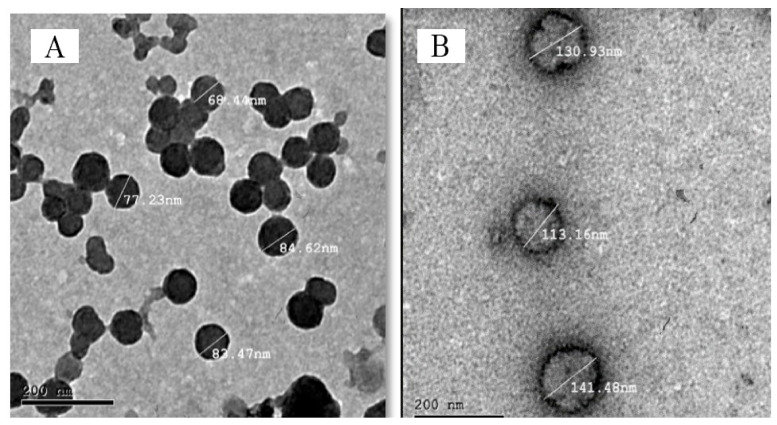
Transmission electron micrograph of ATM-SLNs optimum formula (F9) (**A**) and (F7) (**B**).

Figure 8A,B proved that the two optimum atomoxetine-loaded solid lipid nanoparticles formulae (F7) and (F9), respectively, were spherical in shape with narrow size distribution and no solid lipid nanoparticles with irregular morphology were observed.

### 2.2. Characterization of Lyophilized Nasal Inserts of ATM-Loaded SLNs

#### 2.2.1. Appearance

All Lyophilized nasal inserts of ATM-loaded SLNs formulations had white color, spongy appearance, small cone-like shape and smooth texture, which were useful for placing the solid insert in the nose with minimum irritation [34].

#### 2.2.2. Determination of Drug Content of Lyophilized Nasal Inserts of ATM-Loaded SLNs Formulae

The drug content of atomoxetine measured in the prepared inserts was in the range of 82.5 ± 2.5% to 103.935 ± 3.94%. It was found that the formula of the highest drug content was S8 (103.935 ± 3.94%) and that with the lowest drug content was S1 (82.5 ± 2.5%). It was observed that increasing polymer concentration and glycine (i.e., collapse protecting agent) in the preparation, increased the amount of drug content in the nasal inserts [18]. Moreover, hydroxypropyl methylcellulose (HPMC-K100m) based lyophilized inserts gave higher amount of drug content than (NOVEON) based inserts. On the other hand, lyophilized nasal inserts prepared from stearic acid-based formula (i.e., F7) gave lower amount of drug content than Compritol-based SLNs (i.e., F9) in contrary to the higher amount of entrapped drug in (F7) than in (F9) and that was further explained by the DSC results which showed a larger endothermic peak at 72.88 °C confirming the presence of the stable β’ form of Compritol 888 ATO and atomoxetine was incorporated in the core of Compritol based SLNs and Compritol was in its stable form during formulation processes. The stearic acid-based formula also showed reduction in the principal peak of atomoxetine at 170 °C.

#### 2.2.3. Mass Uniformity of the Solid Nasal Inserts

The average weight of the lyophilized nasal inserts ranged from 52.32 mg ± 4.5% to 94.384 mg ± 4.01% with a weight variation not exceeding 7.5% for each formulation. Therefore, the inserts fall within the acceptable weight variation range according to the European pharmacopeia limits [18].

#### 2.2.4. In-Vitro Drug Release of the Nasal Inserts

**Figure 9:**
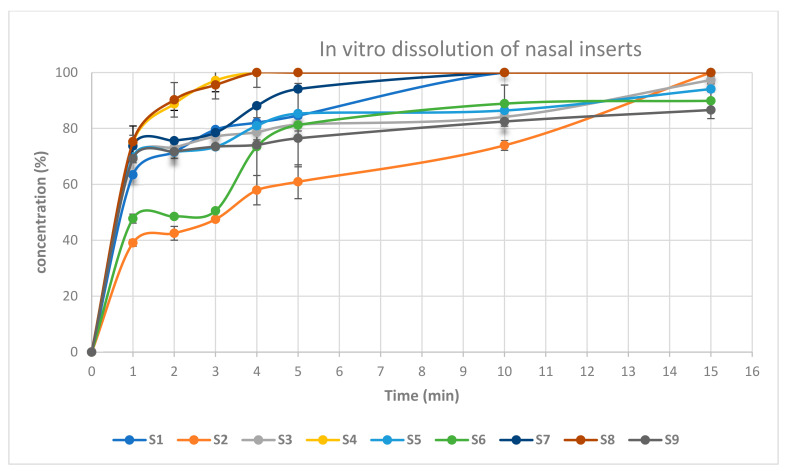
In-vitro drug release of atomoxetine from lyophilized nasal inserts.

Figure 9 shows the cumulative percentage of drug release as a function of time from its nasal inserts. NOVEON AA-1 type nasal inserts containing (0.5%) glycine (S2, S6) had slower release rate than that containing (0.25%) glycine (S1, S5). Moreover, slower release rates were produced upon increasing proportion of NOVEON AA1 used in the formulation [35]. On the other hand, HPMC K100 type nasal inserts containing 0.5% glycine (S4, S8) had faster release rate than that containing 0.25% glycine (S3, S7). The nasal insert formula (S8) showed the highest release rate than other formulae [36]. HPMC is highly swellable, and upon contact with water it diffuses into the polymeric matrix, which results in polymer chains relaxing and expanding. Drugs diffuse out of the system at this point and systems of this type produce mostly fickian diffusion or anomalous transport profiles as shown in Table 4 [37].

#### 2.2.5. Disintegration Time

**Figure 10:**
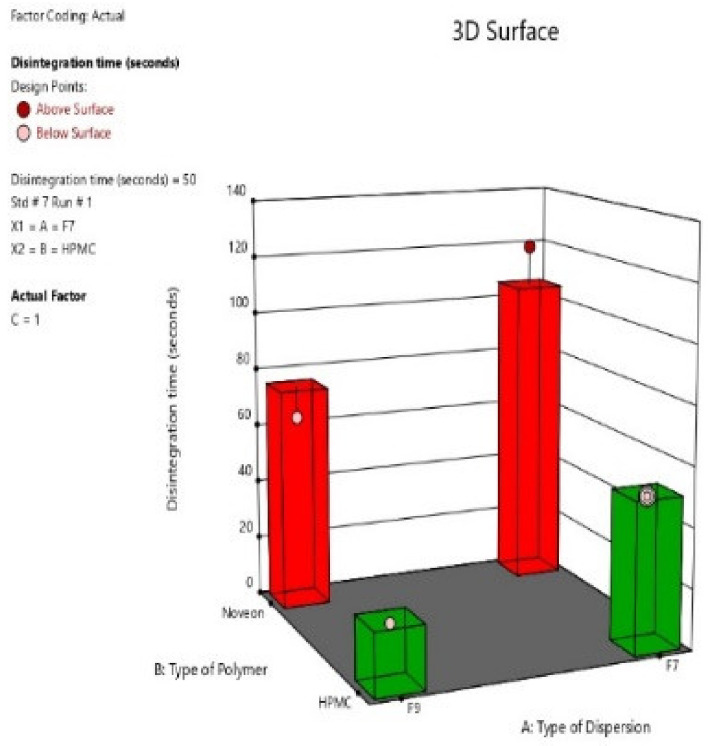
3D-diagram of main effect of type of selected (ATM-loaded SLNs) formula (F7 or F9) (X1) and type of polymer (HPMC K100 or NOVEON) (X2) on disintegration time.

The disintegration time of the eight nasal inserts ranged from 20 s to 125 s. The fastest disintegration time was observed in HPMC type nasal inserts, those being formulae (S4, S8) containing glycine (0.5%) and HPMC K100 (1%). As shown in Figure 10, the slowest disintegration time was observed in Noveon type nasal inserts, those being formulae (S1, S5) containing (0.25%) glycine and Noveon AA1 (0.5%) [38]. On the other hand, HPMC based insert formulated from pure atomoxetine drug solution showed the slowest disintegration time. This could be due to the high surface tension between water and the solid surface of the lyophilized insert and the absence of Surface-active agents (SAA), i.e., Span 60, Pluronic F127 and Poly-vinyl alcohol (PVA) [39].

#### 2.2.6. Muco-Adhesion Strength

**Figure 11:**
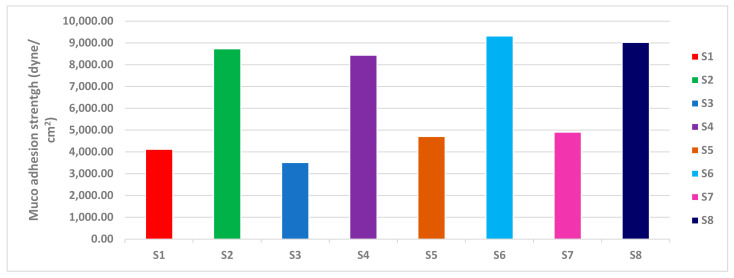
Muco-adhesion strength of lyophilized ATM-loaded SLNs nasal inserts.

The mucoadhesion force for the selected nasal inserts (S4, S8) were (8436.6, 9025.2 dyne/cm^2^), respectively. As for the type of polymer, mucoadhesion force of NOVEON AA1 based nasal inserts showed greater mucoadhesion force than HPMC K100m based nasal inserts. According to the concentration of polymers mucoadhesion force of HPMC 1% relative to HPMC 0.5% (Figure 11) as when the concentration of the polymer increases, the number of penetrating polymer chains per unit volume of the mucin is increased and the interaction between the polymer and mucin become more stable [40].

Polycarbophil (Noveon) is a polyacrylic acid derivative with high molecular weight polymerized in ethyl acetate that contains the tetrafunctional cross-linking agent divinyl glycol. The large number of carboxyl groups in the molecular structure of NOVEON AA1 had been reported to be responsible for binding with the mucosal surfaces through the hydrogen bonding interactions to produce bio-adhesive strength [41]. Therefore, the polymers containing a high density of available hydrogen bonding groups such as NOVEON AA1 linked stronger with mucin. This could explain the results observed in Figure 11.

#### 2.2.7. Optimization of the Lyophilized Nasal Inserts of ATM-Loaded SLNs

**Figure 12:**
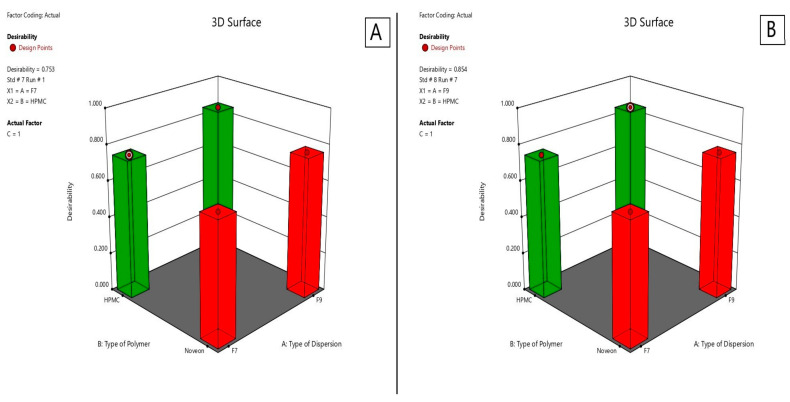
Desirability of S4 lyophilized nasal insert of ATM-loaded SLNs (**A**) and S8 (**B**).

As shown in Figure 12, for selection of the two optimum formulae, the optimization criteria were set to minimize disintegration time and maximize drug content, detachment force and Q15min. As illustrated in Table 5, the two optimum formulae giving the highest numerical desirability values were S8 and S4 with values of 0.854 and 0.753, respectively. As shown in Table 6, the predicted responses for S4 and S8 formulations were detachment force of 9147.92 and 8357.75 (dyne/cm^3^), drug content of 102.91% and 96.52%, disintegration time of 21.25 and 51.25 (s), Q15min of 100.19% and 100%, respectively. Whereas the actual responses were detachment force of 9025.2 and 8436.6 (dyne/cm^3^), drug content of 103.935% and 95.5%, disintegration time of 20 and 50 (s), respectively, and Q15min of 100% for both of them. Hence, lyophilized nasal insert of ATM-loaded SLNs i.e., S4 and S8 could be considered as promising nasal insert formulations. Therefore, they were selected for further investigations.

#### 2.2.8. Differential Scanning Calorimetry (DSC)

**Figure 13:**
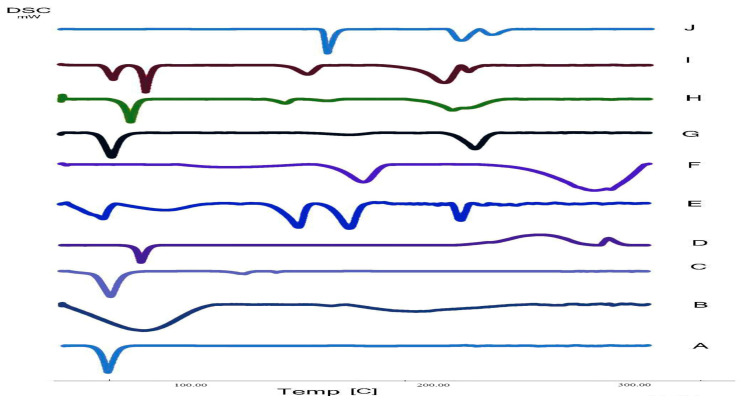
DSC thermogram of **A**: Pluronic F127, **B**: HPMC K100m, **C**: Span 60, **D**: Compritol 888 ATO, **E**: The best formula (S8), **F**: Polyvinyl alcohol (PVA), **G**: Mixture powder of excipients, **H**: The second optimized formula (S4), **I**: Atomoxetine + excipients physical mixture (1:1), **J**: Atomoxetine.

Atomoxetine, HPMC K100m, Compritol 888 ATO, Stearic acid, span 60, Pluronic F127, (1:1) physical mixture of all excipients, atomoxetine: excipients (1:1) physical mixture and the lyophilized optimum ATM-loaded SLNs (S4, S8) DSC thermograms are illustrated in Figure 13. The DSC thermogram of atomoxetine HCl showed a sharply endothermic onset peak at 170 °C [42]. The DSC thermogram of HPMC K100m nasal insert with Compritol 888 ATO (S4) showed the presence of Compritol in its stable polymorph β′ [43]. As, (S4) DSC scan exhibited an endothermic peak at 72.88 °C confirming the presence of the stable β’ form as shown in Figure 13H. Thereafter, atomoxetine was incorporated in the core of compritol based SLNs and Compritol was in its stable form during formulation processes. Additionally, DSC results of S4 and S8 showed compatibility of (ATM) with the other excipients.

#### 2.2.9. Residual Water Content of the Lyophilized Nasal Inserts

Residual moisture content is a key parameter for the stability of atomoxetine and the lipids used in SLNs formulae such as Compritol 888 ATO and stearic acid. The residual water content in NOVEON AA1 based lyophilized nasal inserts (S1, S2, S5, S6) was higher than that of the HPMC K100m based lyophilized nasal inserts. the high glass transition temperature (Tg) of the HPMC K100m was responsible for maintaining the stability of the lyophilizates [44]. The DSC thermogram of HPMC K100m nasal insert with Compritol 888 ATO (S4) showed the presence of Compritol in its stable polymorph β’. As shown in Figure 13, DSC scan of (S4) lyophilized nasal insert of Compritol polymer exhibited an endothermic peak at 72.88 °C confirming the presence of the stable β’ form of Compritol. Additionally, the residual water content of (S4) was 1.4%. As shown in Table 5, Residual water content decreased as polymer concentration increased.

### 2.3. In Vivo Pharmacokinetics Study on Rats

The analytical method utilized for the in vivo study was validated to ensure its linearity, accuracy, and precision. The calibration curves were generated with a determination coefficient of 0.999 both in the plasma and the brain of rats constructed in the range of 10 and 8000 ng/mL. The lower limit of quantification was 10 ng/mL. All other validation results were also fulfilling the acceptance requirements. As shown in Table 7, the most promising results were belonging to the second and third group, where group (2) that was treated with the optimum nasal insert (S8), showed the following results in plasma of rats: 2.99 ± 1.611 h for the half-life (t_1/2_), 130.03 ± 42.63 mg/h/L for the area under the curve (AUC 0–24), and 0.25 h for (T_max_) and the corresponding peak concentration (C_max_) was 108.13 ± 1.884 mg/L. The other optimum lyophilized ATM-loaded SLNs nasal insert (S4), showed the following results in plasma: 1.93 ± 0.19 h for the half-life (t_1/2_), (AUC 0–24) of 121.66 ± 0.19 mg/h/L, and 0.25 h for (T_max_) and the peak concentration was 140.804 ± 56.62925 mg/L.

The (AUC 0–24) and (C_max_) of S8 in brain were 806.18 ± 23.401 and 266.5 ng/mL respectively, while the AUC 0–24 and C_max_ of S4 in brain were 550.21 ± 12.11 and 712.038 ± 96.97 ng/mL, respectively. They were higher than that of the intravenous, oral solution, intranasal insert of pure drug solution and that is owing to the lipophilic nature of the formula S4 due to higher lipid to drug ratio used of Compritol (2:1) atomoxetine used. Therefore, there was better drug permeation through the Blood Brain Barrier. As well as the HLB value of Compritol (HLB = 2) is lower than that of stearic acid (HLB = 14.9) that used in the other formula S8 [45]. Moreover, lyophilized nasal insert (S8) showed the longest mean residence time (MRT) of 12.98 ± 2.75 h which occurred due to the small particle size of stearic acid based solid lipid nanoparticles of (F5) formula which resulted in reducing opsonization and phagocytosis, and increasing brain residence time.

**Figure 14:**
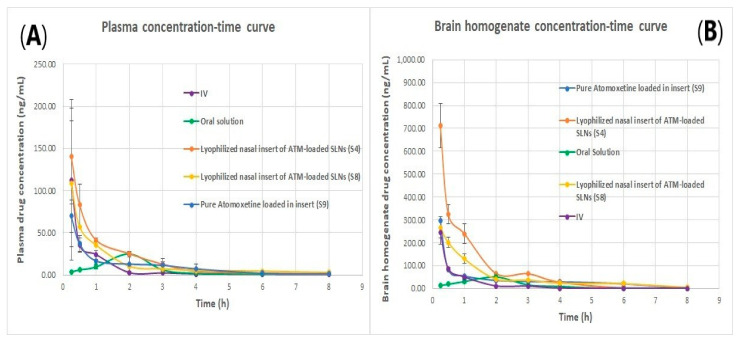
(**A**) plasma concentration time curve of atomoxetine and (**B**) Brain homogenate concentration time curve of atomoxetine.

**Figure 15:**
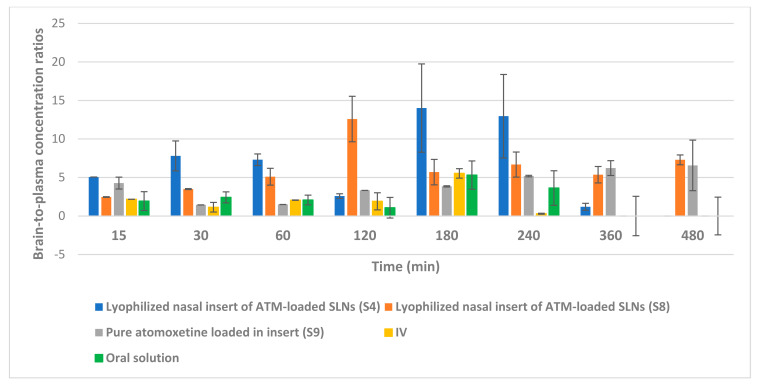
Brain tissue-to-plasma concentration ratios of atomoxetine at 15, 30, 60, 120, 180, 240, 360 and 480 min after administration of the drug by intranasal (IN) route (lyophilized inserts of ATM-loaded SLNs (S4 and S8) and lyophilized insert of pure atomoxetine), intravenous (IV) route and oral route (Atomorelax^®^ oral solution).

As shown in Figure 14, the intranasal (IN) administration of atomoxetine generated a rapid and extensive systemic absorption of the drug similarly to what occurred following IV injection, presenting higher values of C_max_, AUC 0–24 and similar values of T_max_ together with an absolute bioavailability of 163.1 and 166.41% for S4 and S8, respectively. This probably occurred because of the small particle size of the solid lipid nanoparticles. Moreover, atomoxetine is a hydrophilic drug and most drugs that are extensively absorbed through the nasal respiratory epithelium are highly lipophilic and cross it by transcellular mechanisms so, increasing the lipophilicity of the drug by incorporating it into solid lipid nanoparticles will increase the permeation of the drug through the lipophilic blood brain barrier. Additionally, drug transport to the brain was enhanced through solubilization of the endothelial cell membrane lipids, by the surfactants used. Polyvinyl alcohol (PVA), Span 60 and Pluronic f127 incorporated into the prepared ATM-loaded SLNs led to membrane fluidization (surfactant effect). It was recognized that the hydrophobic surfaces of (SLNs) promote protein adsorption. Any shielding of the hydrophobic character of SLNs will thus sterically stabilize them, reducing opsonization and phagocytosis, and increasing brain residence time, blood circulation time and bioavailability. Recognition by the RES was prevented by coating particles with the hydrophilic surfactants (PVA and Pluronic F127) [46]. The two optimized (ATM-loaded SLNs) formulae, i.e., (S4) and (S8) showed higher brain tissue-to-plasma concentration ratios than oral solution, intravenous injection of atomoxetine solution and nasal insert of pure atomoxetine solution which indicates the successful direct nose-to-brain drug delivery, brain deposition and overcoming the systemic clearance of the drug, as shown in Figure 15.

In addition, the brain concentrations of atomoxetine and its pharmacokinetic parameters after intranasal (IN) and intravenous (IV) administration support a direct nose-to-brain delivery of the drug. A Drug targeting efficiency (DTE) percent of 177.42%, 211.3% for S4 and S8, respectively, into the brain, demonstrated that the IN administration of atomoxetine provided another delivery route in addition to the systemic through the BBB, given that a DTE percentage higher than 100% indicates a more efficient brain targeting following IN administration, compared to the systemic administration [47]. It can also be credited to the form of ATM in the intranasal formulation, which contained ATM loaded in SLNs, whereas the intravenous formulation contained ATM in the free form. However, DTE lacks information regarding the fraction of atomoxetine that is directly delivered from the nasal mucosa into the brain. Therefore, DTP was estimated, and revealed that 43.64 and 52.7% for S4 and S8, respectively, of atomoxetine that reached the brain underwent direct nose-to-brain delivery. Negative % DTP indicates more efficient drug delivery to the brain through BBB permeation than direct nose-to-brain routes, whereas the positive values of the % DTE indicate a significant contribution of the direct routes (the olfactory and the trigeminal neural pathway) to the overall brain delivery. S9 nasal insert of free atomoxetine solution yielded a negative %DTP of 9.5% indicating the inability of free atomoxetine to permeate to the brain directly through the olfactory nerve pathway but diffused through the blood-brain barrier confirming the importance of increasing atomoxetine’s lipophilicity by incorporation into solid lipid nanoparticles to enhance its direct nasal transport to the brain.

As shown in Figure 14B, atomoxetine lyophilized nasal inserts achieved a higher brain to plasma concentration ratio in S4 and S8 over the intravenous and oral route of drug administration as well as higher than the free drug solution administered in lyophilized nasal insert.

## 3. Materials and Methods

### 3.1. Materials

Atomoxetine HCl was kindly provided by Multi-APEX for Pharmaceutical Industries (Cairo, Egypt), Risperidone was kindly acquired from Delta Pharma (Cairo, Egypt), polyvinyl alcohol (Grade: High Viscosity, M.Wt: 200,000 Da) and glycine were purchased from ADVENT (Navi-Mumbai, India), stearic acid was purchased from PIOCHEM (6 October City, Giza Governorate, Egypt), compritol^®^ 888 ATO was kindly provided by Gattefossé Co. (Chemin de Genas St Priest, France), span 60, Hydranal^®^ titration solvent, pluronic F127 and HPMC K100 were purchased from Sigma-Aldrich Co. (St. Louis, MO, USA), NOVEON^®^ AA-1 was purchased from Lubrizol Advanced Materials Europe BVBA (Chaussee de wavre, Brussels, Belgium), potassium dihydrogen phosphate, disodium hydrogen phosphate, ammonium acetate, formic acid, methanol and acetonitrile (HPLC grade) were purchased from Merk Co. (North Wales, PA, USA).

### 3.2. Experimental Design

As shown in Table 8 16 formulae were prepared according to a 2^4^ full factorial design by using Design Expert^®^ software program (version 13, Stat-Ease Inc., Minneapolis, MN, USA) by optimization of four independent variables: lipid Type (X1), lipid to Drug ratio (X2), span 60, Pluronic F127 ratio (X3) and probe sonication time (X4). Four responses were determined, those being entrapment efficiency (EE%) (Y1), in vitro drug release after 30 min (Q30min) (Y2), particle size (P.S.) (Y3) and zeta potential (ZP) (Y4). Full factorial design statistical analysis outcomes. The statistical analysis of the factorial design outcomes were performed using Design Expert^®^ (Version 13.0.1, State-Ease Inc., Minneapolis, MN, USA) where statistical evaluations were carried out by comparing between different groups of experiments applying the analysis of variants ANOVA considering the null hypothesis (H0) [48]. If the *p*-value is less than 0.05, the investigated factor is significant as well as the null hypothesis may be rejected [23].

### 3.3. Preparation of Atomoxetine Loaded Solid Lipid Nanoparticles (ATM-SLNs)

As shown in Table 9 ATM-loaded SLNs were prepared by the aid of hot melt emulsification, stirrer and ultrasonication method [23]. First of all phosphate buffer (PH = 5.5) was prepared from a mixture of two solutions [49,50]. Solution (A) was prepared by dissolving 13.61 g of potassium dihydrogen phosphate in one liter of deionized water. Solution (B) was prepared by dissolving 35.81 g of disodium hydrogen phosphate in one liter of deionized water. Phosphate buffer (PH = 5.5) was obtained by mixing 94.6 mL of solution (A) and 3.6 mL of solution (B) [51,52]. Moreover, atomoxetine HCl 20.56 mg (equivalent to 18 mg of Atomoxetine base) and pluronic F127 were dissolved in 1 mL of phosphate buffer (PH = 5.5) containing (1% *w*/*v*) PVA that was previously prepared by mixing 1 g of PVA with 100 mL of phosphate buffer (PH = 5.5) and stirred for 2 h on a magnetic stirrer at 70 °C [53]. Subsequently, in a 100 mL glass beaker the lipid phase composed of stearic acid or compritol 888 ATO and span 60 were heated to 85 °C with moderate mixing by using a high-speed magnetic stirrer. Furthermore, the aqueous phase (composed of atomoxetine, pluronic f127 and PVA (1% *w*/*v*) dissolved in 1 mL of phosphate buffer (PH = 5.5)) was heated to the same temperature of the melted lipid phase for 10 min. The aqueous phase was then added drop wisely to the melted lipid phase and mixed for 10 min at 16,000 rpm using a high-speed magnetic stirrer. Finally, the pre-emulsion was treated with a probe sonicator for 5 or 10 min (1 min pulse on, 1 min pulse off, and 60% pulse frequency). The obtained dispersion was stored at 4 °C for further investigations [54].

### 3.4. Evaluation of the Prepared Atomoxetine HCl Solid Lipid Nanoparticles Dispersion Systems

#### 3.4.1. Entrapment Efficiency (EE%)

One ml of every single formula was transferred to Eppendorf tubes and centrifuged for 30 min at 16,0000 rpm and 4 °C using a cooling centrifuge (Sigma 3 K 30, Germany) [23]. After centrifugation supernatant was discarded and the residue was lysed using methanol and analyzed spectrophotometrically using a UV spectrophotometer (UV SPECORD-210 plus, Analytik Jena, Jena, Germany) at the predetermined wavelength (λmax = 270.5 nm) to determine the amount of entrapped drug [55,56]. Another one ml of each prepared ATM-loaded solid lipid nanoparticles formulae was also dissolved in methanol without separation of residue by using ultracentrifugation and then measured spectrophotometrically to determine the total drug content (Free + entrapped) [57]. The equation used for the determination of entrapment efficiency is as follows [23]:

Percent Entrapment Efficiency (EE%) = (ED/TD) × 100 (1)

i.e., ED is the concentration of entrapped drug and TD is the concentration of the total drug. The results are the mean values of three runs ± SD.

#### 3.4.2. Measurement of the Particle Size (PS), Zeta Potential (ZP) and Polydispersity Index (PDI)

The average particle size diameter in (nm) was measured by Malvern Zetasizer equipment at 25 °C, backscatter detection of 173 °C, and refractive index of 1.330 (Malvern Instruments, Ltd., Malvern, UK). The formulations were diluted with deionized water in a ratio of (1:10), which provided suitable scattering intensity to measure the size of ATM-loaded SLNs. Dynamic light scattering (DLS) was used to measure the PDI of (ATM-SLNs). DLS detects vesicle distribution. The ZP was carried out in deionized water by detecting the electrophoretic mobility of the charged solid lipid nanoparticles in an electrical field, and that indicated their permeation behavior by studying their colloidal property and stability. PDI values less than 0.5 are more common to monodisperse samples, while values larger than 0.7 are common to a broad size (e.g., polydisperse) distribution of particles [57,58]. The results are the mean values of three runs ± SD.

#### 3.4.3. In-Vitro Drug Release Study

The in-vitro drug release of ATM-loaded SLNs was determined using a modified Franz diffusion cell. In simulated nasal conditions, the release rate of atomoxetine was investigated, and the percent of drug release after 30 min (Q30min) was determined. The semi-permeable cellulose membrane (Spectra/Pore dialysis membrane with a 12,000–14,000 molecular weight cutoff) was soaked in phosphate buffer solution (PH = 5.5) for 24 h at 25 °C before being used and attached between the donor and receptor compartments. In a donor compartment, 1 mL sample of the prepared ATM-loaded SLNs was inserted (equivalent to 18 mg of atomoxetine). The media was 100 mL phosphate buffer (pH 5.5) applied in the receptor compartment, with continuous stirring at 50 rpm with the aid of a magnetic stirrer at 37 °C. At predefined time intervals, 1 mL sample was collected and filtered using 0.45 syringe membrane filter paper, and the quantity of atomoxetine released was measured spectrophotometrically using UV spectrophotometer (UV-1601 PC spectrophotometer, Shimadzu, Kyoto, Japan) at the predetermined λmax (270.5 nm). The samples were taken at 5, 10, 15, 30, 45, 60, 120, 180, 240, 300, 360, 420 and 480 min and immediately replaced with a fresh phosphate buffer (PH 5.5) medium [19,23,56].

#### 3.4.4. Release Kinetics

The mechanism of drug release was determined by applying various mathematical models, including the Korsmeyer peppas model, Higuchi model, first order, and second-order kinetics In this study, the model with the highest coefficient of determination (R2) was identified [32].

#### 3.4.5. Statistical Optimization of ATM-Loaded SLNs Formulae

Design Expert^®^ software program (version 13, Stat-Ease Inc., Minneapolis, MN, USA) was used to determine the desirability function, which allowed for the statistical analysis of all responses at the same time. The desirability function was used to determine the best two formulae. The optimization criteria were chosen to provide two formulations with the lowest particle size and the highest entrapment efficiency, zeta potential (in an absolute value) and Q30min. The two trials with the highest desirability rating were chosen [24].

#### 3.4.6. Transmission Electron Microscope (TEM)

Transmission electron microscope was used to determine morphological characteristics of the two optimized atomoxetine solid lipid nanoparticle dispersion systems. The results could be used to understand the in vitro-drug release profile and the in vivo pharmacokinetics of the two optimized formulae. A drop of each of the two optimized formulae (F7) and (F9) was diluted with distilled water and spotted on two different carbon-coated copper grids. Using phosphotungstic acid (2% *w*/*v*), the spotted drops were negatively stained and then allowed to dry at room temperature. Finally, the stained samples were visualized using transmission electron microscopy. (JEM 1230, Jeol, Tokyo, Japan) [58].

#### 3.4.7. Experimental Design Construction of Lyophilized Nasal Inserts of ATM-Loaded SLNs

As shown in Table 10 eight formulations had been formulated based on a 23 full factorial design using Design Expert^®^ software (version 13, Stat-Ease Inc., Minneapolis, MN, USA). By optimization of three independent variables, those being the type of ATM-loaded SLNs optimized formula (X1), polymer type (X2) and polymer concentration (X3). There were four responses: Drug content(Y1), amount of drug released in 15 min (Q15min) (Y2), Disintegration time (Y3), and Muco-adhesion strength (Y4). We applied the analysis of variants ANOVA to the factorial design outcomes using Design Expert^®^ (Version 13.0.1, State-Ease Inc., Minneapolis, MN, USA). Statistical evaluations were conducted by comparing various groups of experiments based on the null hypothesis (H0). If the *p*-value is less than 0.05, a factor is significant and the null hypothesis may be rejected [59,60].

#### 3.4.8. Preparation of Lyophilized Nasal Inserts of Atomoxetine Solid Lipid Nanoparticles (ATM-SLNs)

As shown in Table 11, eight inserts were prepared by lyophilization of solutions of the matrix former HPMC K100m or NOVEON AA1, glycine (Collapse protecting agent) and the required amount of ATM-loaded SLNs dispersion to give 10 mg atomoxetine per insert. First, glycine was dissolved in approximately one-third of the required amount of distilled water (10 mL of water) in a glass beaker with the use of a magnetic stirrer adjusted at 37 °C ± 0.5 °C and 600 rpm. To obtain uniform gel, the needed weight of HPMC or NOVEON was slowly sprinkled over glycine solution, while stirring continuously. The PH of the obtained gel solution was measured and adjusted with tri-ethanolamine to be maintained at PH = 5.5. Gel solution containing 12 mg/mL atomoxetine base was transferred to 1.5 mL Eppendorf tubes and freeze dried for 24 h using a Virtis Advantage freeze drier (Virtis, New York, NY, USA), with pre-set cycle stages; freezing) for 8 h, reducing temperature from −30 to −60 °C in 10 °C increments), primary drying (18 h increasing temperature from 10 to 22 °C in 5 °C increments, with chamber pressure decreasing from 100 to 40 mTorr) [18,61,62].

### 3.5. In-Vitro Characterization of Lyophilized Inserts ATM-Loaded SLNs

#### 3.5.1. Appearance

The appearance and physical properties, as the shape, color and texture of each prepared insert were characterized [18].

#### 3.5.2. Assay of Drug Content

In a 100mL volumetric flask, 1 insert was first disintegrated with 5 mL of deionized water and then 30 mL of methanol was added and the flask put in the water bath sonicator for 15 min until the complete dissolution of the nasal insert. The concentration of the drug was determined spectrophotometrically using a UV spectrophotometer (UV-1601 PC spectrophotometer, Shimadzu, Kyoto, Japan) at the predetermined λ_max_ (270.5) [55,56].

#### 3.5.3. Uniformity of Weight

According to the European Pharmacopeia 2020 guidelines for tablets, the test was conducted. Each of the 20 nasal inserts from each formula were weighed separately. The average weight of inserts was calculated. The inserts pass the test if no more than two inserts deviate from the average weight by more than 7.5% from and no insert differ by more than twice that percentage [18,63].

#### 3.5.4. In-Vitro Dissolution of the Nasal Inserts

The drug release profile of each insert was studied. Each lyophilized insert of ATM-loaded SLNs (equivalent to 10 mg atomoxetine) was tested using the USP dissolution apparatus I (Agilent Technologies 708-DS, Santa Clara, CA, USA). The receiver vessel contained 250 mL phosphate buffer (PH 5.5) at 37 ± 0.5 °C with rotating basket speed of 50 rpm. An aliquot of (1 mL) was withdrawn at predetermined time intervals over 1 h. Sampling time at 1, 2, 3, 4, 5, 10, 15, 20, 30, 45 and 60 min and immediately replaced with a fresh phosphate buffer (PH 5.5) medium. The withdrawn aliquots were spectrophotometrically analyzed using UV spectrophotometer (UV SPECORD-210 plus, Analytik Jena, Jena, Germany), at the pre-determined wavelength (λ_max_ = 270.5).

#### 3.5.5. Disintegration Time

One nasal lyophilized insert was put in a beaker containing 200 mL of water at 15–25 °C, it should be disintegrated within 3 min. The test was repeated on 5 more nasal lyophilized inserts from the same batch. The formula is considered to comply with the test when all the 6 inserts had disintegrated in the predetermined time [18].

#### 3.5.6. Determination of Mucoadhesion Strength of the Nasal Inserts

Mucoadhesion strength of each lyophilized nasal insert was determined by using a laboratory with a 250 mL beaker on the right side and a 100 g weight suspended from a thin steel wire on the left side. A 250 mL inverted beaker served as the basis for the hanging weight when the balance was restored equalized. Normal saline (0.9%) was used to clean two fresh slices of rabbit small intestine. One was fastened with cyanoacrylate adhesive to the bottom of the 100 g weight, while the other was affixed to the base of an upside-down 250 mL beaker. One insert was put between the two sections and left for five minutes to ensure proper contact. After that, water was infused into the beaker on the right side at a set rate of 13–15 drops per minute using an infusion device. The beaker’s weight kept rising until the two slices on the opposing side were just separated from one another. The following equation was used to acquire the mucoadhesive force (also known as the detachment stress) (dyne/cm^2^) using the following equation. The results are the mean values of three runs ± SD [32].

Detachment stress (dyne/cm^2^) = (m × g)/A (2)

where (A) is the area of the rabbit’s small intestine (area of contact), (m) is the weight of water, and (g) is the acceleration due to gravity taken as 981 cm [57,64,65].

#### 3.5.7. Residual Water Content of the Lyophilized Inserts

By using Karl Fischer titration, the residual water content of the nasal inserts was determined after lyophilization process. A known volume of dry methanol (Sigma-Aldrich, Bornem, Belgium) was used to reconstitute the lyophilized insert and was left to equilibrate for 15 min. Next, a known volume of this solution was removed from the vial via a syringe, injected in the titration vessel of a Mettler Toledo V30 volumetric Karl Fischer titrator (Schwerzenbach, Switzerland), and titrated with Hydranal^®^ titration solvent (Sigma-Aldrich, Bornem, Belgium). The moisture content of the dry methanol was determined to subtract from the result before testing the lyophilized insert sample [44,66].

#### 3.5.8. Optimization of the Lyophilized Nasal Inserts of ATM-Loaded SLNs

As shown in Table 10, for selection of the two optimum lyophilized nasal inserts of ATM-loaded SLNs formulae, the optimization criteria were to minimize disintegration time and maximize drug content, detachment force and Q15min. The two nasal inserts with the highest desirability value obtained from Design Expert^®^ software program (version 13, Stat-Ease Inc., Minneapolis, MN, USA) will be chosen for further investigations in DSC study and in vivo study on rats [24].

#### 3.5.9. Differential Scanning Calorimetry (DSC)

The thermal assessment of atomoxetine, HPMC K100m, Compritol 888 ATO, stearic acid, span 60, pluronic F127, physical mixture of all excipients with (1:1) molar ratio, physical mixture of atomoxetine and all other excipients with (1:1) molar ratio and the two optimum lyophilized nasal insert formulae (S4, S8) was carried out by DSC (Shimadzu Corp., Kyoto, Japan) standardized with indium. The samples (3–5 mg) were placed in aluminum pans and then heated from 25 to 350 °C at a rate of 10 °C/min, using an empty pan as a reference under nitrogen flow [42,67].

### 3.6. In-Vivo Pharmacokinetics Study

Wistar male albino rats were attained from the House of Animal of Faculty of Veterinary medicine, Cairo University, Giza, Egypt. Animal care and housing procedures were carried out in accordance with the guidelines of the Research Ethics Committee (REC) of Cairo University. The protocol of research was recognized by the Animal Care Committee of the National Research Center (Cairo, Egypt) and was accepted by the Ethics Committee (PI 2813 in November 2020).

#### 3.6.1. Administration and Sampling

Wistar male albino rats (average weight: 200 ± 20 g) were randomly divided into six groups (parallel group design). Group (1) was injected with atomoxetine (3 mg/kg, intraperitoneally). Group (2) was given the optimum lyophilized nasal insert (S8), Group (3) was given another optimum lyophilized nasal insert formula (S4), Group (4) was given lyophilized nasal insert of pure drug solution coded (S9), Group (5) was given the marketed oral solution (Atomorelax^®^ 20 mg/5 mL oral solution). Finally, Group (6), (Control group) was given normal saline 0.9% orally. The rat dose was 3 mg/kg in all the groups except for control group [68]. The dose was calculated based on the following equation [69]:

(3) $$\mathrm{Human}\;\mathrm{dose}\;(\mathrm{mg}/\mathrm{kg})=\mathrm{Animal}\;\mathrm{dose}\;(\mathrm{mg}/\mathrm{kg})\;\times \left(\frac{\mathrm{Animal}\;\mathrm{Km}}{\mathrm{Human}\;\mathrm{Km}}\right)$$

where Km is the conversion factor and equal 37 and 6 for human and rat, respectively [70].

The rats are housed under conditions of 14h light, 10h darkness, at a temperature of (23 ± 2 °C) and a relative humidity of (60 ± 10%). They will be kept on a standard pellet chow and allowed water ad libitum (7% simple sugars, 3% fat, 50% polysaccharide, 15% protein (*w*/*w*), energy 3.5 kcal/g). The rats are housed and kept in an environment with 14 h of light and 10 h of darkness, as well as a temperature of (23 ± 2 °C) and relative humidity of (60 ± 10%). They will be kept on a standard pellet chow and allowed unlimited access to water and regular diet (7% simple sugars, 3% fat, 50% polysaccharide, 15% protein (*w*/*w*), energy 3.5 kcal/g). The rats were positioned in a supine position with a head angle of 90 degrees during nasal administration and even after dosing for 2 min to prevent drainage of the insert. Nasal administration was carried out using polyethylene tube (inner diameter: 0.1 mm), fitted to Hamilton syringe [71]. The intravenous injection was given intraperitoneal in the animal’s lower right quadrant of the abdomen to avoid damage to the internal organs. The oral drug solution was given with a needless syringe to the mouth of rats [16]. At each time interval (0.25, 0.5, 1, 2, 3, 4, 6, 8 and 24 h), 12 rats, two from each group, were sacrificed. From each rat blood samples were collected and the brain was separated. Blood samples were centrifuged at 3000 rpm for 15 min using a cooling ultracentrifuge (Beckman, Fullerton, Canada) to separate plasma. The separated brains, on the other hand, were dried and rinsed in saline solution. Whole brains and plasma samples were both stored at −70 °C until analysis [19].

#### 3.6.2. Sample Preparation

Before analysis, brain tissues were homogenized in deionized water to obtain a 50% *w*/*v* dispersion using a Heidolph DIAX 900 homogenizer (Heidolph, Germany). Calibration standards of atomoxetine in plasma and brain samples were prepared at a concentration range of 1, 10, 50, 100, 300, 500, 700, 900, 1000 ng/mL, prepared as follows: 50 µL of IS and atomoxetine reference standard solutions were spiked to 450 µL plasma blank mixed with K-EDTA and vortexed for 30 s. Quality control samples of LLOQ (10 ng/mL), Low-QC (30 ng/mL), Medium QC-A (200 ng/mL), Medium QC-B (400 ng/mL) and High-QC (800 ng/mL) were prepared. 50 µL of 5 µg/mL IS in the aliquot of 0.5 mL Plasma was spiked, vortexed for 30 s, protein precipitation was carried out by adding 2 mL Acetonitrile, samples vortexed for 4 min, centrifuged at 5000 rpm for 5 min, then 1 mL of upper clear supernatant was diluted with 0.5 mL Water, and 5 μL was injected into the LC–MS/MS system.

#### 3.6.3. LC/MS/MS Analysis

A previously reported, accurate and sensitive LC/MS/MS analysis technique was utilized for the drug analysis in plasma and brain samples [72]. A liquid chromatographic system (Shimadzu, Tokyo, Japan) equipped with degasser (DGU-20A3), solvent delivery unit (LC-20AB) with an auto-sampler (SIL-20 AC) was utilized to inject 10 μL samples at a flow rate of 0.4 mL/min using a 3-min gradient method, starting at 90% acetonitrile and 10% 0.1%formic acid in H_2_O + 10 mM ammonium acetate to a C18 column (Hypersil Gold C18 2.1 × 50 mm × 5 μM). The mass spectrometer (API-4000, SCIEX, Framingham, MA, USA) was equipped with turbo ion spray operated in positive mode and set at 5000 V. The common parameters between the drug and its internal standard were set at 10 psi, 20 psi, 40 psi and 3 psi for curtain, nebulizer, auxiliary and collision gases, respectively. On the other hand, the specific parameters, including collision energy, de-clustering potential, entrance potential and collision exit were set at 10.5 V, 50 V, 10 V and 9 V for atomoxetine and 24 V, 67.7 V, 10 V and 14 V for risperidone. Multiple reaction monitoring detected the transition of atomoxetine precursor from 256.100 *m*/*z* to 148.100 *m*/*z* and risperidone precursor from 237.300 *m*/*z* to 194.00 *m*/*z*. Data were handled using Analyst software version 1.6 (SCIEX, Framingham, MA, USA) [72].

#### 3.6.4. Pharmacokinetics Parameters

The pharmacokinetic parameters of atomoxetine following nasal administration of the best two formulae (S4 and S8), the intravenous drug solution, the oral solution, and the nasal administration of lyophilized insert of free drug solution were determined using non-compartmental analysis. The peak maximal concentration (C_max_) and its corresponding time (T_max_) were directly determined from the brain and plasma concentration-time curves. Moreover, Linear trapezoidal method was used to calculate the areas under the curves from zero to the last time (AUC 0) and to infinity (AUC 0-inf.). The measured concentrations were transformed to natural logarithms before calculation of the elimination half-life (t_1/2_), elimination rate constant (K_el_) and mean residence time (MRT). The drug partitioning into the brain was determined through calculation of the brain targeting efficiency (BTE%) using the following equation [73]:

BTE% = ((BIN/PIN)/(BIV/PIV)) × 100(4)

where BIN and PIN are the area under the curves from zero to infinity in brain homogenate (B) and plasma (P), respectively, after intranasal (IN) administration of either the nasal insert of drug solution or the optimized lyophilized atomoxetine solid lipid nanoparticles in nasal insert formula. On the other hand, BIV and PIV are the area under the curves from zero to infinity in brain homogenate (B) and plasma (P), respectively, after intravenous (IV) administration.

Moreover, the drug transport percentage (DTP%) was calculated using the following equation [73]:

DTP% = ((BIN − BX)/BIN) × 100(5)

where BX = (BIV/PIV) × PIN.

Additionally, the absolute bioavailability of atomoxetine after nasal administration was calculated using the following equation [74]:

F (absolute bioavailability) = (D i.v. × AUC i.n.)/(D i.n. × AUC i.v.)(6)

where (D) is the dose of atomoxetine, (AUC i.n. 0–∞) is the area under the concentration-time curve after intranasal administration in the plasma of rats and (AUC i.v. 0–∞) is the area under the concentration time curve after intravenous administration in plasma of rats.

#### 3.6.5. Statistical Analysis

One-way analysis of variance (ANOVA) will be used to compare between different groups of samples and a concurrent control using GraphPad Instat version 3 software (GraphPad Instat Software, Inc., San Diego, CA, USA) [23]. Additionally, the data will be expressed as the means ± standard deviation. A *p*-value less than 0.05 will be considered to indicate a significant difference between groups.

## 4. Conclusions

The current study showed that the prepared intranasal lyophilized inserts of (ATM-SLNs) showed an enhanced absolute bioavailability, higher brain targeting efficiency (DTE%) and higher direct transport percentage (DTP%) of atomoxetine over the marketed oral solution (Atomorelax^®^), intraperitoneal drug solution and the pure drug solution in nasal insert. Additionally, an efficient nose-to-brain delivery of atomoxetine was achieved by increasing atomoxetine’s lipophilicity by incorporation into solid lipid nanoparticles. Both Compritol and stearic acid contributed to the formation of small particle size solid lipid nanoparticles that successfully delivered the drug to the target site (The Brain). Lyophilized nasal insert of Compritol-based solid lipid nanoparticles achieved (DTP%) of 52.7% while lyophilized nasal insert of stearic acid -based SLNs achieved (DTP%) of 43.64%. Additionally, HPMC based lyophilized nasal inserts enhanced the mucoadhesion of the insert onto the nasal mucosal surface. The proposed lyophilized nasal inserts of ATM-loaded SLNs represents an alternative dosage form to the oral marketed drug solution (Atomorelax^®^) by bypassing the BBB and delivering atomoxetine directly to the brain. As well as, increasing the stability of the drug and decreasing the metabolism by the liver.

## Figures and Tables

**Table 1:** Output data of the 2^**4**^ full factorial analysis of ATM-loaded SLNs.

ATM-Loaded SLNs Formulation	A: (X1) Lipid Type	B: (X2) Lipid to Drug Ratio	C: (X3) Span 60: PF127 Ratio	D: (X4) Probe Sonication Time	(Y1) EE%	(Y2) PS (nm)	(Y3) PDI	(Y4) ZP (mV)	(Y5) Q30min	Desirability
F1	Stearic acid	2:1	1:3	10 min	59.12 ± 9.38	496.9 ± 45.21	0.435 ± 0.11	−20.0 ± 0	36.615%	0.469
F2	Stearic acid	2:1	1:3	5 min	53.89 ± 8.13	459.1 ± 46.8	0.276 ± 0.122	−19.4 ± 1.3	32.5%	0.444
F3	Stearic acid	2:1	3:1	10 min	46.5085 ± 7.09	700.5 ± 25.88	0.351 ± 0.1	−21.3 ± 1.3	37.77%	0.410
F4	Stearic acid	2:1	3:1	5 min	43.9575 ± 3.35	426.8 ± 62.89	0.291 ± 0.101	−18.8 ± 1.3	27.11%	0.000
F5	Compritol 888	2:1	1:3	10 min	90.5725 ± 2.8	445.3 ± 130.9	0.478 ± 0.1	−17 ± 1.6	68.875%	0.513
F6	Compritol 888	2:1	1:3	5 min	71.025 ± 4.5	445.3 ± 89.181	0.359 ± 0.05	−16.3 ± 0.5	63.9075%	0.600
F7	Compritol 888	2:1	3:1	10 min	83.0575 ± 4.1	435.2 ± 167.3	0.377 ± 0.1	−28.4 ± 0.212	86.75%	0.654
F8	Compritol 888	2:1	3:1	5 min	75.9925 ± 0.5	660.6 ± 133.7	0.261 ± 0.1685	−17.1 ± 0.79	69.65%	0.504
F9	Stearic acid	1:2	1:3	10 min	55.1975 ± 7.83	392.1 ± 43.5	0.222 ± 0.132	−16.5 ±1.11	54.963%	0.628
F10	Stearic acid	1:2	1:3	5 min	54.5 ± 1	320.9 ± 110.81	0.299 ± 0.04	−8.52 ± 0.7	50.5%	0.000
F11	Stearic acid	1:2	3:1	10 min	41.1375 ± 1.8	390.9 ± 194.013	0.532 ± 0.03	−17.6 ± 0.8	69.397%	0.438
F12	Stearic acid	1:2	3:1	5 min	38.0425 ± 1.85	719.8 ± 26.5	0.516 ± 0.03	−17.9 ± 0.75	60.3%	0.489
F13	Compritol 888	1:2	1:3	10 min	73.6625 ± 15.1	487.9 ± 68.4	0.493 ± 0.03	−15.3 ± 0	72.663%	0.470
F14	Compritol 888	1:2	1:3	5 min	62.79 ± 2.52	390.9 ± 66.6	0.559 ± 0.01	−18.8 ± 0.62	70.02%	0.543
F15	Compritol 888	1:2	3:1	10 min	59.979 ± 5.6	936.7 ± 229.6	0.658 ± 0.03	−16.8 ± 0.82	91.08%	0.000
F16	Compritol 888	1:2	3:1	5 min	52.82 ± 3.8	718.9 ± 221.1	0.611 ± 0.04	−20.1 ± 3.2	87.01%	0.429

**Note:** Data represented as mean ± SD (*n* = 3). **Abbreviations**: **EE%**: Entrapment Efficiency; **PS**: Particle Size; **ZP**: Zeta Potential; **PDI**: Polydispersity Index; **Q30min**: Amount of drug released after 30 min; **ATM**: Atomoxetine; **SLNs**: Solid Lipid Nanoparticles; **NA**: Not available.

**Table 2:** Experimental runs, independent variables, and measured responses of the 2^4^ full factorial experimental design of ATM-loaded SLNs.

Responses	EE (%)	PS (nm)	ZP (mV)	Q30min (%)
Adequate precision	21.4	10.18	373.31	21.1408
Adjusted R^2^	0.9646	0.8446	0.9998	0.9623
Predicted R^2^	0.8793	0.4694	0.9967	0.8713
Significant factors	X1, X2, X3, X4	X1, X3, X4	X1, X2, X3, X4	X1, X2, X3, X4
Observed value of the two optimum ATM-SLNs formulae (F7) and (F9)	83.06 and 55.20	795.60 and 392.10	−28.4 and −16.50	86.75 and 54.96
Predicted value of two optimized ATM-SLNs formulae (F7) and (F9)	83.29 and 54.97	801.52 and 374.89	−28.39 and −16.51	84.25 and 51.98
Model fitting	Analysis of variance table partial sum of squares-type III for all responses.			

**Abbreviations: EE%**: Entrapment Efficiency Percent; **PS**: Particle Size; **ZP**: Zeta Potential; **Q30min**: amount of drug released after 30 min; **ATM-SLNs**: solid lipid nanoparticles of atomoxetine.

**Table 3:** Kinetic release parameters of atomoxetine from the solid lipid nanoparticles formulations.

Formulation Code	Zero Order (R^2^)	First Order (R^2^)	Diffusion	Korsmeyer Peppas	(n)	Mechanism
F1	0.8695	0.7049	0.919	**0.9553**	0.374	Fickian diffusion
F2	0.8779	0.5846	**0.9652**	0.9133	-	Diffusion
F3	0.9647	0.9043	**0.9854**	0.9603	-	Diffusion
F4	0.8563	0.4359	**0.9458**	0.7818	-	Diffusion
F5	0.9631	0.8325	**0.9851**	0.9828	-	Diffusion
F6	0.921	0.4021	**0.9672**	0.6972	-	Diffusion
F7	0.4682	0.3918	0.649	**0.799**	0.1224	Fickian diffusion
F8	0.7531	0.5905	0.8884	**0.9208**	0.3709	Fickian diffusion
F9	0.8854	0.8349	0.963	**0.9649**	0.2481	Fickian diffusion
F10	0.8609	0.8073	**0.9461**	0.9398	-	Diffusion
F11	0.8449	0.779	0.9471	**0.9808**	0.1996	Fickian diffusion
F12	0.8645	0.7997	0.9584	**0.9819**	0.2723	Fickian diffusion
F13	0.7412	0.6471	0.8858	**0.9588**	0.2801	Fickian diffusion
F14	0.7613	0.642	0.8974	**0.9498**	0.328	Fickian diffusion
F15	0.4702	0.4122	0.6506	**0.8178**	0.1487	Fickian diffusion
F16	0.7704	0.6771	0.9059	**0.9654**	0.2546	Fickian diffusion

**Note:** Bold values identify the release kinetic model, (n): release exponent.

**Table 4:** Kinetic release parameters of atomoxetine from the solid lyophilized nasal inserts.

Formulation Code	Zero (R^2^)	First (R^2^)	Diffusion	Korsmeyer Peppas	(n)	Mechanism
S1	0.4585	0.4366	0.6546	**0.8504**	0.1123	Fickian diffusion
S2	0.6105	0.5741	0.7909	**0.9179**	0.2715	Fickian diffusion
S3	0.6496	0.6338	0.8217	**0.9339**	0.1029	Fickian diffusion
S4	0.1695	0.1628	0.2895	**0.4902**	0.0473	Fickian diffusion
S5	0.6413	0.6169	0.8153	**0.9274**	0.1031	Fickian diffusion
S6	0.5165	0.46	0.6762	**0.7944**	0.1884	Fickian diffusion
S7	0.4153	0.404	0.6018	**0.7954**	0.0834	Fickian diffusion
S8	0.1765	0.1684	0.2994	**0.5002**	0.0462	Fickian diffusion
S9 (lyophilized nasal insert of free drug solution)	0.7426	0.7319	0.8791	**0.9273**	0.1088	Fickian diffusion

**Note:** Bold values identify the release kinetic model, (n): release exponent.

**Table 5:** Characterization of lyophilized ATM-loaded SLNs nasal inserts.

Formulation Code	Drug Content (%)	Q15min (%)	Mass Uniformity (mg)	Disintegration Time (s)	Residual Water Content (%)	Muco-Adhesion Strength (dyne/cm^3^)	Desirability
S1	82.5 ± 2.5%	100 ± 0%	76.8 ± 3.9%	100	2.87%	4120.2 ± 39.43	0.393
S2	86.5 ± 1.5%	100 ± 0%	94.4 ± 4%	120	0.5%	8730.9 ± 61.36	0.656
S3	91.375 ± 1.125%	97.324 ± 1.4%	79.8005 ± 4.42%	65	1.88%	3510.5 ± 140.21	0.407
S4	95.5 ± 1%	100 ± 0%	91.34 ± 6.4%	50	1.4%	8436.6 ± 39.43	0.753
S5	98.55 ± 5.55%	94.118 ± 1.9%	52.3225 ± 4.5%	100	4.32%	4708.8 ± 61.36	0.543
S6	100.5 ± 0.5%	89.9 ± 6.4%	59.395 ± 3.3%	65	2.033%	9319.5 ± 39.425	0.747
S7	93.665 ± 2.84%	100 ± 0%	57.6 ± 1.71%	30	0.60%	4905.39 ± 162.145	0.590
S8	103.935 ± 3.94%	100 ± 0%	67.75 ± 3.6%	20	2.62%	9025.2 ± 61.36	0.854

**Note:** Data represented as mean values ± SD (n = 3). Abbreviations: **Q15min**: amount of drug released after 30 min.

**Table 6:** Experimental runs, independent variables, and measured responses of the 2^3^ full factorial experimental design of lyophilized nasal insert of ATM-loaded SLNs.

Responses	Drug Content (%)	Q15min (%)	Disintegration Time (s)	Detachment Force (dyne/cm^2^)
Adequate precision	7.1530	19.81	9.5751	31.9
Adjusted R^2^	0.8365	0.9792	0.8441	0.990
Predicted R^2^	0.495	0.8103	0.0.6436	0.9771
Significant factors	NA	NA	A, B.	A, C.
Observed value of the two optimum nasal insert formulae (S4) and (S8)	95.5% and 103.94%	100%	50 s and 20 s	8436.6 and 9025.2
Predicted value of the two optimum nasal insert formulae (S4) and (S8)	96.52% and 102.91%	100.19%	51.25 s and 21.25 s	8357.75 and 9147.92
Model fitting	Analysis of variance table partial sum of squares-type III for all responses.			

**Abbreviations: Q15min**: amount of drug released after 15 min.

**Table 7:** Pharmacokinetic parameters of atomoxetine in plasma and brain homogenate tissues following its intranasal (IN) route (lyophilized inserts of ATM-loaded SLNs (S4 and S8) and lyophilized insert of pure atomoxetine), intravenous (IV) route and oral route (Atomorelax^®^ oral solution).

Pharmaco-Kinetics Parameters	Plasma				Brain					
	Group 1 (IV)	Group 2 (S8)	Group 3 (S4)	Group 4 (S9)	Group 5 (Oral Solution)	Group 1 (IV)	Group 2 (S8)	Group 3 (S4)	Group 4 (S9)	Group 5 (Oral Solution)
C_max_ (ng/mL)	112.59 ± 95.107	108.13 ± 1.884	140.804 ± 56.62925	69.572 ± 19.5265	25.184 ± 2.282	244.894 ± 19.9	266.5 ± 41.9	712.038 ± 96.97	297.05 ± 53.59	49.04 ± 9.8
T_max_ (h)	0.25	0.25	0.25	0.25	2	0.25	0.25	0.25	0.25	2
K_el_ (h^−1^)	0.5361 ± 0.27%	0.232 ± 0.08	0.3592 ± 0.035	0.21 ± 0.094	0.851 ± 0.5	0.215 ± 0.05	0.081 ± 0.017	0.568 ± 0.4	0.341 ± 0.04011	0.58 ± 0.03
t_1/2_ (h)	1.293 ± 0.9%	2.99 ± 1.611	1.93 ± 0.19	3.3024 ± 1.8	0.815 ± 1.7115	3.37 ± 0.6	8.9 ± 1.9	1.22 ± 0.462	2.061 ± 0.24	1.198 ± 0.061
MRT (h)	1.9 ± 1.3%	4.32 ± 2.32	2.78 ± 0.27	4.77 ± 2.6	1.18 ± 2.47	4.9 ± 0.521	12.98 ± 2.75	1.55 ± 0.66	2.974 ± 0.35	1.73 ± 0.09
AUC_(0–24 h)_ (ng∗h/mL)	73.86 ± 5.9	130.03 ± 42.63	121.66 ± 38.9	96.31 ± 15.5	49.16 ± 0.18	135.84 ± 17.25	806.18 ± 23.401	721.44 ± 79.6	267.6312 ± 14.58115	91.87 ± 8.8
AUC(0–∞) (ng∗h/mL)	155.45 ± 26.73	253.46 ± 18.3	258.69 ± 23.01	245.2632 ± 31.01	143.512 ± 5.838	238.33 ± 19.73	821.12 ± 81.58	703.7 ± 1.9	343.41 ± 35.41	191.8646 ± 8.7463
DTE (%)		211.3	177.42	91.33						
DTP (%)		52.7	43.64	−9.5						
F (%)		163.1	166.41							

**Abbreviations: (F)**: Absolute intranasal bioavailability; **AUC_(0–24 h)_**: Area under the concentration time-curve from time zero to 24 h; **AUC(0–∞)**: Area under the concentration time-curve from time zero to infinite; **C_max_**: Maximum peak concentration; **DTE (%)**: Drug targeting efficiency percentage; **K_el_**: Apparent elimination rate constant; **MRT**: mean residence time; **t_1/2_**: Apparent terminal elimination half-life; **T_max_**: time to achieve the maximum peak concentration and **DTP (%)**: direct transport percentage. Data are the mean values ± SD.

**Table 8:** 2^3^ Full factorial design for the optimization of atomoxetine-loaded solid lipid nanoparticles (ATM-SLNs).

Factors (Independent Variables)	Levels	
	Low Level (−1)	High Level (+1)
A:X1: Lipid type	Stearic acid (HLB = 14.9)	Compritol 888 ATO (HLB = 2)
B:X2: Lipid-to-drug ratio	1:2 i.e., (9 mg/mL: 18 mg/mL)	2:1 i.e., (36 mg/mL: 18 mg/mL)
C:X3: Co-surfactant ratio (span 60): (pluronic F127)	1:3 i.e., (1.25 mg/mL: 3.75 mg/mL)	3:1 i.e., (3.75 mg/mL: 1.25 mg/mL)
D:X4: Probe sonication time	5 min	10 min
Responses (Dependent variables)	Desirability constraints	
Y1: EE%	Maximized	
Y2: PS (nm)	Minimized	
Y3: ZP (mV)	Maximized (absolute value)	
Y4: Q30min	Maximized	

**Abbreviations: ATM**: atomoxetine; **EE**: entrapment efficiency; **PS**: particle size; **SLNs**: solid lipid nanoparticles; **ZP**: zeta potential; **HLB**: Hydrophilic-Lipophilic Balance; **N.B**: Conditions of probe sonication; Temperature 40 °C, Pulse Frequency 60%, Cycles number 5 cycles.

**Table 9:** The composition of atomoxetine-loaded solid lipid nanoparticles (ATM-SLNs).

ATM-Loaded SLNs Formulation Code	Lipid Type		Surfactant PVA (1%) min	Co-Surfactant Mixture (0.5%)		Span 60: PF127 Ratio	Lipid: Drug Ratio	Probe Sonication Time	
	Stearic Acid (mg/mL)	Compritol 888 (mg/mL)		Span 60 (mg/mL)	PF127 (mg/mL)			5 min	10 min
F1	36	-	10	1.25	3.75	1:3	2:1	-	on
F2	36	-	10	1.25	3.75	1:3	2:1	on	-
F3	36	-	10	3.75	1.25	3:1	2:1	-	on
F4	36	-	10	3.75	1.25	3:1	2:1	on	-
F5	-	36	10	1.25	3.75	1:3	2:1	-	on
F6	-	36	10	1.25	3.75	1:3	2:1	on	-
F7	-	36	10	3.75	1.25	3:1	2:1	-	on
F8	-	36	10	3.75	1.25	3:1	2:1	on	-
F9	9	-	10	1.25	3.75	1:3	1:2	-	on
F10	9	-	10	1.25	3.75	1:3	1:2	on	-
F11	9		10	3.75	1.25	3:1	1:2	-	on
F12	9		10	3.75	1.25	3:1	1:2	on	-
F13		9	10	1.25	3.75	1:3	1:2	-	on
F14		9	10	1.25	3.75	1:3	1:2	on	-
F15		9	10	3.75	1.25	3:1	1:2	-	on
F16		9	10	3.75	1.25	3:1	1:2	on	-

**Abbreviations: ATM-loaded SLNs**: atomoxetine loaded solid lipid nanoparticles; **PVA**: Polyvinyl Alcohol; **PF127**: Pluronic flakes 127.

**Table 10:** 2^3^ Full factorial design for the optimization of lyophilized nasal inserts of ATM-loaded SLNs.

Factors (Independent Variables)	Levels	
	Low Level (−1)	High Level (+1)
X1: Type of (ATM-SLNs) formula	F7 (compritol-based SLNs)	F9 (stearic acid-based SLNs)
X2: Polymer type	HPMC K100m	NOVEON^®^ AA-1 USP polycarbophil
X3: Polymer concentration	NOVEON (0.25%) and HPMC K100 (0.5%)	NOVEON (0.5%) and HPMC K100 (1%)
Responses (Dependent variables)	Desirability constraints	
Y1: Assay of drug content (%)	Maximize	
Y2: Q15min (%)	Maximize	
Y3: Disintegration time (seconds)	Minimize	
Y4: Muco-adhesion strength (dyne/cm^2^)	Maximize	

**Abbreviations: ATM-SLNs**: atomoxetine loaded solid lipid nanoparticles; **HPMC**: Hydroxypropyl methylcellulose; **Q15min**: amount of drug released after 15 min.

**Table 11:** Composition of the prepared lyophilized nasal inserts of ATM-loaded SLNs.

Nasal Insert Formulation Code	Drug Concentration (%)	Polymer (Matrix Former) Type and Concentration		Glycine Concentration	
S1	1%	-	NOVEON 0.25%	0.25%	-
S2	1%	-	NOVEON 0.5%	-	0.5%
S3	1%	HPMC K100 0.5%	-	0.25%	
S4	1%	HPMC K100 1%	-		0.5%
S5	1%	-	NOVEON 0.25%	0.25%	-
S6	1%	-	NOVEON 0.5%	-	0.5%
S7	1%	HPMC K100 0.5%	-	0.25%	
S8	1%	HPMC K100 1%	-		0.5%
S9 (Lyophilized nasal insert of pure drug solution)	1%	HPMC K100 1%	-	-	0.5%

**Abbreviations: HPMC:** Hydroxypropyl methylcellulose; **NA:** Not available.

## Data Availability

Data are contained within the article.
